# Collective memory in primate conflict implied by temporal scaling collapse

**DOI:** 10.1098/rsif.2017.0223

**Published:** 2017-09-06

**Authors:** Edward D. Lee, Bryan C. Daniels, David C. Krakauer, Jessica C. Flack

**Affiliations:** 1 Department of Physics, Cornell University, 142 Sciences Drive, Ithaca, NY 14853, USA; 2 ASU–SFI Center for Biosocial Complex Systems, Arizona State University, Tempe, AZ 85287, USA; 3 Santa Fe Institute, 1399 Hyde Park Rd, Santa Fe, NM 87501, USA

**Keywords:** conflict, pigtailed macaques, collective behaviour, collective memory, scaling collapse

## Abstract

In biological systems, prolonged conflict is costly, whereas contained conflict permits strategic innovation and refinement. Causes of variation in conflict size and duration are not well understood. We use a well-studied primate society model system to study how conflicts grow. We find conflict duration is a ‘first to fight’ growth process that scales superlinearly, with the number of possible pairwise interactions. This is in contrast with a ‘first to fail’ process that characterizes peaceful durations. Rescaling conflict distributions reveals a universal curve, showing that the typical time scale of correlated interactions exceeds nearly all individual fights. This temporal correlation implies collective memory across pairwise interactions beyond those assumed in standard models of contagion growth or iterated evolutionary games. By accounting for memory, we make quantitative predictions for interventions that mitigate or enhance the spread of conflict. Managing conflict involves balancing the efficient use of limited resources with an intervention strategy that allows for conflict while keeping it contained and controlled.

## 1. Introduction

In biology, conflict plays a central role in structuring interactions among components, whether genes, cells or individuals. Conflict occurs when components have only partially aligned interests [1,2]. Examples of conflict include fights among group members in animal and human societies, infection, immune responses and even autoimmunity, in which conflict arises when an immune response targets self instead of a pathogen. Conflict growth—the spread of conflict from a small number of antagonistic or infected components to many—has been modelled in a diversity of systems as a contagion process [3], where the resulting conflict duration (e.g. length of fights or duration of infection [4]) can vary over several orders of magnitude.

A growing body of work suggests that a benefit of small, contained conflicts is that they allow components to test and refine strategies at relatively a low cost [5,6]. This can facilitate adaptation and innovation [7]. Large and long conflicts have been associated with system instability and increased component mortality [3,8–10]. Whether there are key factors influencing conflict size and duration across a range of biological systems is not yet understood, although in the specific case of primate conflict some factors contributing to conflict size have been identified [11,12].

We investigate the dynamics of conflict duration using an animal society model system—a group of captive, socially housed pigtailed macaques (*Macaca nemestrina*, *N* = 64) at the Yerkes National Primate Research Center (see electronic supplementary material, §0.1.1). Here, conflicts can manifest as fights. A fight starts when one individual threatens or attacks a second individual. The total number of individuals participating in a given fight ranges from 2 to 35, with third-parties becoming involved through intervention and redirected aggression (electronic supplementary material, §0.1.2).

Fights have clear, operationally defined starting times and endpoints and generally only one fight is active at a given time (electronic supplementary material, §0.1.2). This produces a time series of fights separated by peaceful periods. The time series was measured at the resolution of seconds, collected over a period of roughly four months, and contains approximately 1000 cycles of peace and conflict. We observe a wide range of conflict durations (1–840 s) and peace durations (2–5570 s). We use these data to infer and characterize the dynamics underlying the durations of conflict and peace.

We find that peaceful periods are characterized by a ‘first to fail’ process (as in reliability theory [13]) in which the duration of peace depends only on the waiting time for the first pair of individuals to begin fighting. The distribution of peaceful periods is exponential, consistent with pairs independently choosing to begin fights, and the likelihood of remaining in the peaceful state does not depend strongly on the recent past (electronic supplementary material, §0.2).

We find conflict, on the other hand, displays increased variance in duration, consistent with strong correlations between the aggressive interactions that occur within a fight. This suggests a ‘first to fight’ mechanism: the beginning of the conflict influences the duration of consecutive pairwise interactions, and the end of fighting retains a *collective memory* of the start. Collective memory is encoded in the aggregate interactions in a conflict (we return at the end of Results and in Discussion to how this collective memory arises and whether it implies individual memory). Distributions of fight duration are consistent with a universal lognormal distribution under a simple scaling transformation that accounts for fight size. In contrast to common models of conflict growth [14], fight duration cannot be explained using a simple memoryless contagion process.

## 2. Results

**Figure 1.**
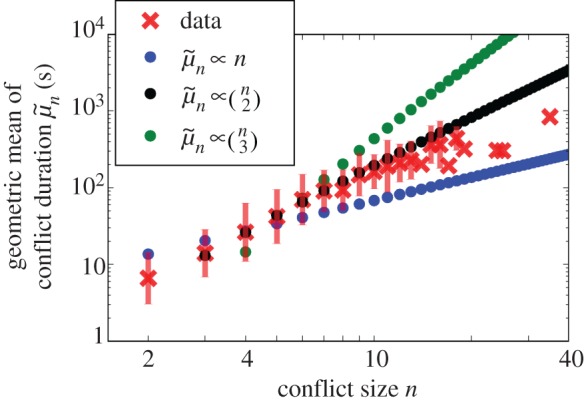
Conflict duration scales superlinearly with conflict size. Observed geometric means of conflict durations (red circles) grow proportionally to the number of potential pairwise interactions 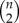 (black). Fits proportional to 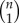 and 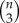 are shown in blue and green, respectively. Error bars are twice the geometric standard deviation. A similar scaling relationship appears in the duration of interstate human conflict as shown in electronic supplementary material, figure S9. (Online version in colour.)

A simple contagion model of conflict growth corresponds to a branching process in which each new individual joining the fight induces others to join with some average probability *p* (electronic supplementary material, table S1). As long as the average fight size *μ* = 3.8 remains small relative to the system size *N* = 64, the probability of recruiting another individual is small (*p* ≪ 1) and the cascade of aggressive interactions connecting individuals forms a tree. A natural null model for how conflicts progress in time is that each aggressive interaction adds, on average, some constant duration to the fight, and consequently the mean conflict duration should grow linearly with *n*, the number of individuals participating. If these aggressive interactions were to overlap in time, fight duration would grow at most linearly with *n*. As we show in figure 1, the mean fight duration grows superlinearly with fight size *n*. Notably, similar superlinearity of duration with conflict size appears in interstate human wars (electronic supplementary material, §0.6). This rules out a simple contagion model in which tree-like growth describes the duration of aggressive interactions.

**Figure 2.**
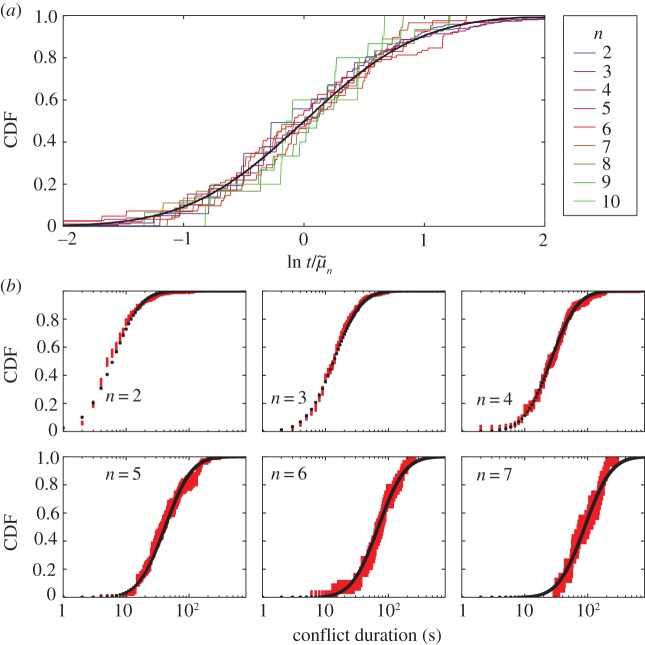
Scaling collapse of conflict duration. (*a*) Scaling collapse for conflict duration of fights of sizes 2–10, accounting for 97% of the data. Universal lognormal curve fit to the shown data overlaid in black. All shown duration distributions except for *n* = 2 are statistically indistinguishable from the discretized lognormal. (*b*) Detailed comparisons for a few conflict sizes (red with 90% CI) with the universal lognormal curve discretized by seconds (black). (Online version in colour.)

To compare fight duration distributions across fight sizes, we must account for the longer time scale of larger fights. We rescale by the geometric means 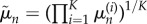, and all distributions collapse onto a single universal curve with a common geometric standard deviation 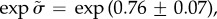 as shown in figure 2 (electronic supplementary material, §0.3). This corresponds to a coefficient of variation 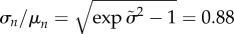. That is, fights of a given size have fluctuations in duration with magnitude approximately 88% of the mean across all observed fight sizes. The collapse implies multiplicative scaling similar to Weber's Law, though the size of fluctuations is too large to be explained simply as errors in temporal perception.^1^

This collapse implies that the distributions behave like translated versions of one another in logarithmic space. A natural form for such distributions is the lognormal [18,19]:2.1
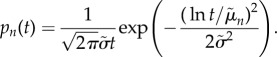
We show a universal lognormal fit to the collapsed distributions in figure 2. The lognormal closely matches the shape of duration distributions for every fight size, and it is statistically indistinguishable from the data (discrete KS-test *K* < 0.2, *p* > 0.5) except for fights of size 2 (*K* = 0.09, *p* < 0.01).^2^

The superlinear growth of the mean in figure 1 implies that the fight duration per individual grows larger as more individuals become involved. Fights involve interactions between multiple individuals and so we look for a terminating time scale associated with the number of potentially interacting subgroups of size *γ*. This corresponds to geometric mean duration that grows as 
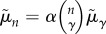
, where *n* ≥ *γ* and *α* refers to the fraction of subgroups of size *γ* that are realized, each extending the conflict by a typical amount of time 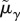. By maximum likelihood, we find *γ* = 2 and *α* = 0.66, and we compare this fit to the means in figure 1 alongside the analogous fits with *γ* = 1 and *γ* = 3. According to the best fit, the typical number of aggressive interactions is approximately 2 for *n* = 3, in agreement with observation. Triadic interactions typically consist of 1 to 2 aggressive interactions, and additional interactions consist largely of redirected aggression towards a third party, an impartial intervention by a third party, or a partial intervention in which the intervener directs affiliative behaviour or aggression towards participants (electronic supplementary material, §0.1.2). For *n* > 3, conflict duration continues to be proportional to approximately two-thirds of the number of realizable pairwise interactions, suggesting that further participants interact with not just one past participant, but a larger subset.^3^

If we assume that conflicts consist of sequential pairwise interactions, the scaling collapse displayed in figure 2 suggests that interactions in any given fight are correlated in time. A perfect scaling collapse implies both that the geometric variance is constant and the (arithmetic) standard deviation grows linearly with the geometric mean, 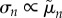 (electronic supplementary material, §0.3). In the opposite case with uncorrelated, independent units, the scaling is expected to be different, 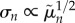, as would be the case with the arithmetic mean in the context of the Central Limit Theorem (electronic supplementary material, table S1). Plotting the variance against the mean (electronic supplementary material, figure S4), we find the relation is significantly different from the Central Limit Theorem prediction and that correlations are important [20].

These results indicate that interaction durations are correlated within each conflict. Hence any model that we construct must be constrained by the observations of both the superlinear scaling of the mean and the scaling of the standard deviation that implies significant correlations between pairwise interactions.

**Figure 3.**
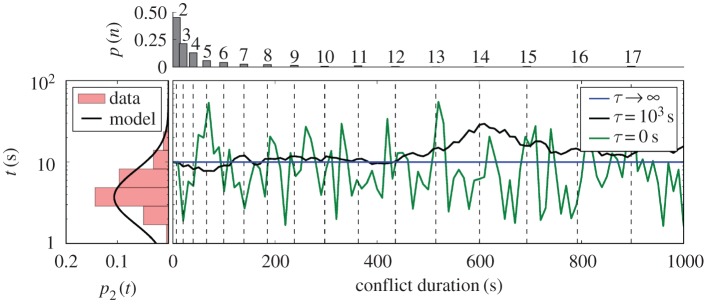
Example trajectories from a diffusion-aggression model. Duration of a conflict with three or more participants is the sum of the durations of pairwise interactions. The initial interaction is sampled from a lognormal distribution for conflicts of size 2, *p*_2_(*t*) (left) from equation (2.1). The following interactions are generated by a random walk with decay time *τ*. In the case where 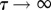, all consecutive pairwise interactions are perfectly correlated (blue). As *τ* gets smaller (black then green), the interaction durations decorrelate faster. The dashed vertical lines show our prediction for the typical duration for a fight of size *n* as predicted by 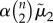 (figure 1), and we show the corresponding probability of observing a conflict of size *n* (top). (Online version in colour.)

To capture these features, we propose a simple model, represented in figure 3. A fight in the model consists of a series of pairwise interactions with the duration set by a randomly walking aggression state. Temporal correlations correspond to constraining the random walker to remain close to the previous intensity of aggression.

We characterize the memory of the random walker about the previous intensity of aggression with a diffusion constant *D* that controls the rate at which correlations decay between sequential interactions [21]. In the limit in which the duration of each interaction is independent, 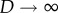, the total length of the fight is a sum of uncorrelated random variables, and we recover *σ*_*n*_ ∝ *μ*^1/2^_*n*_. In the opposite limit, 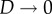, all interactions are perfectly correlated, *σ*_*n*_ ∝ *μ*_*n*_, and the rescaling procedure is exact. In this limit, a large fight is simply a rescaled version of a fight of size 2 (electronic supplementary material, table S1). As we change *D* to vary the amount of correlation between subsequent interactions, we cross over between these two limits. From the scaling collapse, we expect that *D* ≪ 1 [22]. Thus, we picture a fight as a sequence of interactions that wander in aggression space with a diffusion constant that determines the rate at which the system forgets how the fight started.

**Figure 4.**
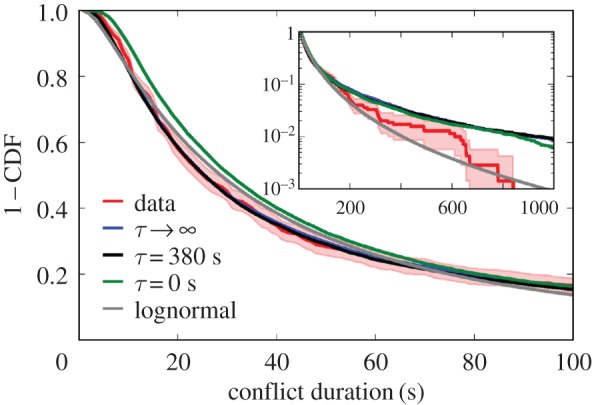
Comparisons among aggregate distributions of conflict duration. Diffusion models with long correlation times *τ* (blue, black) capture the distribution of conflicts with three or more participants (red with 90% bootstrapped CIs). Especially at small, typical durations, they compare favourably to a simple lognormal fit (grey), and to uncorrelated interaction durations (green). KS statistics are *K* = 0.03 (*p* = 0.3) for 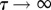, *K* = 0.03 (*p* = 0.5) for *τ* = 380 s, *K* = 0.1 (*p* ≤ 10^−5^) for *τ* = 0 s, and *K* = 0.06 (*p* < 10^−2^) for the lognormal.

Given that fight durations scale with the number of pairwise interactions, we use the statistics of the observed distribution of conflicts of size 2 to generate the corresponding distribution for larger fight sizes. We sample interactions from a lognormal fit to the distribution of pairwise fights and concatenate 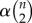 temporally correlated, pairwise interactions for a conflict of size *n*. By varying *D*, we find that we fit the observed distributions well when the typical decorrelation time *τ* > 270 s (figure 4; electronic supplementary material, S5 and S6).

The lower bound on the correlation time at *τ* > 270 s corresponds to strong correlations between sequential agonistic interactions (electronic supplementary material, table S1). The typical conflict with three or more participants is 60 s long, and over 96% of those conflicts are shorter than 270 s, showing that nearly all observed fights retain correlated interactions over their entire duration (electronic supplementary material, §0.4). This observation suggests that temporal correlations are strong over the course of a single fight and that the length of the first pairwise incident significantly influences the evolution of the conflict over time.

Correlated interactions are likely to persist over the course of conflict due to cognitively mediated or emotionally mediated memory for past interactions. This collective memory could be reducible to individual memory if it is caused by individuals participating in multiple dyads within the same conflict and behaving similarly in each. Alternatively, irreducible collective memory occurs when individual decisions to join and remain in a fight are a function of these decisions by others.^4^ Different mechanisms for producing irreducible collective memory make different demands on individual-level memory: individuals may remember the duration or severity of (i) all previous dyads within the fight, (ii) only the initial bout or (iii) only the immediately previous dyad within the fight.

To understand how conflict can be controlled, a crucial question to answer is how long and how large an ongoing conflict might become. For example, high-power individuals called policers regulate conflict by intervening in fights in some macaque societies [24,25]. Monitoring conflicts, however, consumes time and attention and interventions carry the risk of injury and this risk increases with fight size [24]. Knowing how long a conflict is likely to last given the number of individuals involved, or how big it will become given how long it has already lasted, would help intervening individuals decide how to distribute their interventions.

We can estimate the probability that more individuals will join an ongoing fight and the probability that the fight will have a given duration. With the joint distribution of conflict size and duration *p*(*t*, *n*) = *p*(*t*|*n*)*p*(*n*), the probability that a fight might be extended by time Δ*t* with Δ*n* additional members given that we have observed *n*_0_ participants after *t*_0_ elapsed time is an application of Bayes' theorem, which yields (electronic supplementary material, §0.5)2.2
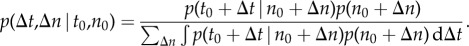


**Figure 5.**
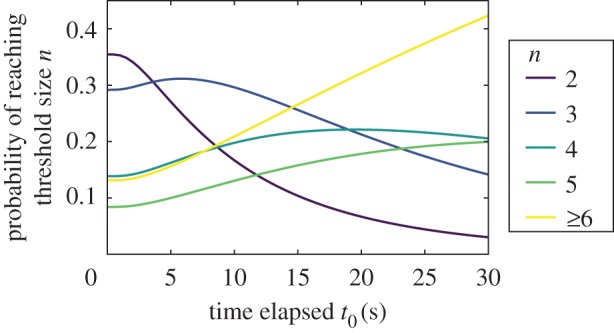
Probabilities of conflict size growth for a dyadic fight as a function of time elapsed *t*_0_ as in equation (2.2). It is most probable that the conflict will not grow in the first few seconds, but it quickly decays as the probability that it grows by at least one more member dominates between 4 and 15 s. At 15 s, the most probable outcome is a fight of size 6 or greater. Monitoring and intervention strategies can be developed depending on, for example, knowledge that interventions are ineffective after a critical fight size. More details given in electronic supplementary material, figures S10 and S11. (Online version in colour.)

In figure 5, we show how the expected total fight size changes the longer a conflict with two individuals lasts. Since the probability that the dyadic conflict remains dydadic decays exponentially, one strategy for minimizing conflict size favours earlier intervention: it is more probable that the fight will grow the longer it has lasted. If the probability of a successful intervention terminating a fight becomes very difficult at, for example, a size of 6, our model suggests that an effective intervention time is approximately 15 s. After this point, the probability of the fight reaching size 6 is high. Similarly, if duration has important functional consequences, our model suggests the policer or conflict manager can estimate duration by monitoring the conflict size (electronic supplementary material, §0.5).

We cannot, however, use this analysis to identify the intervention strategies adopted by individuals in this system. The fights analysed here already contain policing and other types of interventions. Some interventions terminated the fight or reduced its intensity, others had no apparent effect (except to increase fight size), and some exacerbated fight severity (electronic supplementary material, §0.1). This is a typical feature of fights in this system. Hence, this analysis reveals how an intervener could strategically apportion additional interventions given these events.

## 3. Discussion

Simple branching processes have been proposed to underlie many growth and contagion processes in biology and social science [14,26]. We observe that for a primate conflict, a standard branching process does not naturally capture the temporal dynamics since (i) conflict durations are proportional to the number of pairs of individuals in a fight and not the number of individuals, and (ii) bouts within a conflict are influenced by how long previous dyadic events last [27]. Superlinear scaling suggests that conflict resolution requires time not merely for each agitated individual to become inactive but for a large fraction of pairwise relationships between involved individuals to be separately resolved. The duration correlation suggests that for fighting pairs the duration of fight bouts in the population is a salient conflict feature and that conflict growth is a function of the collective memory of a group.

Collective memory, when it is not entirely reducible to individual memory, implies a long time scale associated with repeated interactions between participants in a conflict [28]. In other words, the history of the conflict affects the game under consideration including payoffs. The idea that a conflict is shaped by a persistent social memory is, of course, not new. It was captured, for example, by von Clausewitz when he wrote that ‘war is *simply* the continuation of politics by other means’ [29]—the initial disagreement is present in every conflict. Our results show that there is a quantitative basis for this idea and hence that many of the frameworks typically applied to conflict, such as Markovian games with iterated interactions, may be inappropriate, because they assume a short time scale.

The universal scaling of conflict durations in this society demonstrates the key role of memory in conflict growth. This scaling has implications for conflict management strategies, which are likely to reflect trade-offs between the costs and benefits of intervention and monitoring. In systems with collective memory, a relatively simple strategy to control the conflict growth is to target initial interactions. This would be efficient if sustained conflict monitoring is costly or interventions are ineffective in larger conflicts.

In systems in which conflict managers can reliably estimate the relationship between conflict duration and the number of participants, we predict that interventions will be targeted selectively towards specific fight sizes or fight durations. This type of *targeted* conflict intervention tailored to conflict features stands in contrast to conflict management mechanisms that control conflict systemically at regular intervals [30]. We might expect such regular temporal control when managers cannot respond reliably to specific conflicts.

## Supplementary Material

Supplementary materials

## Endnotes

1 In perceptual tasks, this is related to the Weber ratio: if fights were to end simply by individuals stopping after some fixed perceived duration, then we expect a corresponding temporal Weber ratio roughly the size of the observed coefficient of variation. Temporal Weber ratios are typically 0.5 or less in both animals and humans, depending on the particular task and the duration of the interval being estimated [15–17]. Our measured value of 0.9 is significantly larger, suggesting variation beyond that implied by individual temporal estimation.

2 The fact that we can distinguish size 2 durations as being distributed in a more complicated way than lognormal could be related to increased statistical resolution at this size (fights of size 2 are most frequent), difficulties in measurement of very short durations (fights of size 2 have very short mean length *μ*_2_ = 2.2 s), or lack of convergence to lognormal due to a smaller number of contributing independent factors. Nevertheless, the lognormal distribution is a close fit to the probability distribution for pairwise conflicts.

3 As we discuss in the electronic supplementary material, larger fights typically cover more area of the compound which means that as participants drop out of fights, the probability of re-engagement later in the fight becomes less likely than for small fights covering a smaller area of the compound. Moreover, larger fights by definition are more diverse and if individuals are less likely to direct aggression at unfamiliar participants, then we should expect that in large fights there should be a decrease from small fights in the number of interactions. This may account for failure of our model to capture the slower growth of the mean conflict duration for larger fight sizes as in the right side of figure 1. However, there is insufficient statistical evidence to support a more complicated model that fits the handful of points deviating at large conflict sizes.

4 Such irreducibility is reflected in measures of synergy applied to conflict participation data [23].

## Data accessibility

Data used for this paper are available online at https://github.com/eltrompetero/conflict/tree/master/collective_memory_interface. Contact J.C.F. for questions about data.
