# Supplementary material for: Collective memory in primate conflict implied by temporal scaling collapse
Source: J R Soc Interface. 2017 Sep 6;14(134):20170223. doi: 10.1098/rsif.2017.0223 (PMC5635334; doi:10.1098/rsif.2017.0223)
Supplement: Supplementary materials [file rsif20170223supp1.pdf]

Supplementary Materials for "Collective memory in primate conflict implied by temporal scaling collapse" in *J. R. Soc. Interface* (2017)

Edward D Lee,[1*] Bryan C Daniels,[2] David C Krakauer,[2,3] Jessica C Flack[2,3]

[1*]Department of Physics, Cornell University, 142 Sciences Drive, Ithaca, NY 14853

[2]ASU–SFI Center for Biosocial Complex Systems, Arizona State University, Tempe, AZ 85287

[3]Santa Fe Institute, 1399 Hyde Park Rd, Santa Fe, NM 87501

*To whom correspondence should be addressed; E-mail: edl56@cornell.edu.

0.1 Empirical Methods & Study System Description

0.1.1 Study system

In this section, we provide details on our empirical study system. The data were collected by JCF in 1998 from a large group of captive pigtailed macaques (*Macaca nemestrina*) socially-housed at the Yerkes National Primate Center in Lawrenceville, Georgia. Pigtailed macaques (*1*) are indigenous to south East Asia and live in multi-male, multi-female societies characterized by female matrilines and male group transfer upon onset of puberty. Pigtailed macaques breed all year. Females develop swellings when in Œstrus. Macaque societies more generally are characterized by social learning at the individual level, social structures that arise from nonlinear processes and feed back to influence individual behavior, frequent non-kin interactions and multiplayer conflict interactions (reviewed in (*2*)).

The study group contained $n = 64$ non-infant individiuals (adults, subadults and juveniles) and 84 individuals in total. The study group had a demographic structure approximating wild populations and subadult and adult males were regularly removed to mimic emigration occur-

ring in wild populations. All individuals, except 8 (4 males, 4 females), were either natal to the group or had been in the group since formation. The group was housed in an indoor-outdoor facility, the outdoor compound of which was 125 x 65 ft.

Data on social dynamics and conflict were collected from this group over a stable, four month period. Operational definitions are provided below in SI Sec. 0.1.2.

0.1.2 Operational definition of a fight

Fight: includes any interaction in which one individual threatens or aggresses a second individual. A conflict was considered terminated if no aggression or withdrawal response (fleeing, crouching, screaming, running away, submission signals) was exhibited by any of the conflict participants for *two minutes* from the last such event. A fight can involve multiple individuals. Third parties can become involved in pairwise conflict through intervention or redirection, or when a family member of a conflict participant attacks a fourth-party. Fights in the data set analyzed here ranged in size from 2 to 35 individuals, counting only the socially-mature animals. Fights can be represented as small networks that grow and shrink as pairwise and triadic interactions become active or terminate until there are no more individuals fighting under the above described two minute criterion. In addition to aggressors, a conflict can include individuals who show no aggression or submission (*e.g.* third-parties who simply approach the conflict or show affiliative / submissive behavior upon approaching, and recipients of aggression who show no response to aggression (typically, threats) by another individual). Because conflicts involve multiple actors, two or more individuals can participate in the same conflict but not interact directly.

In this study only information about the number of participants in a fight and duration are used. Only fights that included two or more socially-mature individuals were used in the analysis; the data includes $N = 1086$ such fights. We do not consider internal aspects of the fight,

such as who does what to whom, except for the order of each individual's first involvement in the fight (used to estimate time-ordered conditional probabilities for use in the dynamical branching process model). Time data were collected on fight onset and termination but only total duration data are used in these analyses.

0.1.3 Data collection protocol

The data were collected by JCF using all-occurrence sampling using a voice recorder and digital stop watch and working from an observation tower. All individuals were visible during data collection. Observations were uniformly distributed over the hours of 1100 to 2030.

0.2 Peace is different from conflict.

Pigtailed macaque conflict is characterized by long peaceful periods, with mean duration $\sim$400 s, that are interrupted by brief periods of conflict, with mean duration $\sim$40 s. We compare the distributions of peace and fight durations in Fig. S1a. Both span multiple orders of magnitude, but are distinct in shape. We emphasize this difference in Fig. S1b by rescaling each distribution by its standard deviation. Fight durations have a fatter tail quantified by a skewness of 5.1 compared to 3.3 for peace (KS statistic of 0.40, $p < 10^{-9}$).

The near-linear behavior of the cumulative distribution function (CDF) of peace durations on a log-linear scale as in Fig. 0.2 suggests an exponentially distributed process, corresponding to a probability density function for duration t of

$$p(t) = \Lambda e^{-\Lambda t}, \tag{1}$$

The maximum likelihood estimate of Λ corresponds to a mean duration of peace of $1/\Lambda = 414$ s. The simple exponential is a close fit to the data (Kolmogorov-Smirnov statistic of 0.07 with $p < 10^{-4}$), but there is a small systematic deviation at the tail.

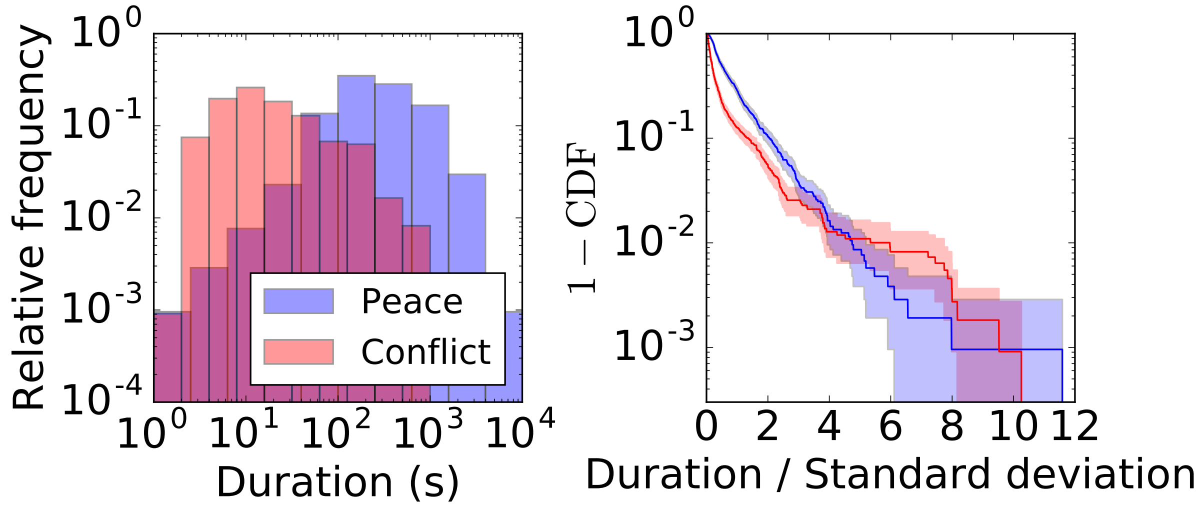

Figure S1: Comparison of peace and conflict durations. (left) Distributions of peace and conflict durations. (right) Complementary cumulative distribution function of peace and conflict durations rescaled by standard deviation for comparison. The distributions are different as captured by the KS statistic of 0.40 ($p < 10^{-9}$). The difference in the decay of the tails is reflected in the skewness—defined as the normalized third moment $\langle((t-\mu)/\sigma)^3\rangle$—of 3.3 and 5.1 for peace and fights, respectively.

The exponential distribution is a signature of peaceful periods with a fixed rate of decay, but we know that the rate of fight outbreak fluctuates between days and even over the course of the day. One strong pattern is that peaceful periods tend to be shorter later in the day as in Fig. 0.2. Motivated by this observation, we propose a simple description that Λ is uniformly distributed over some interval $(\Lambda - \Delta\Lambda, \Lambda + \Delta\Lambda)$, a mixture of exponentials weighted evenly over varying Λ. We find with maximum likelihood $((\Lambda + \Delta\Lambda)^{-1}, (\Lambda - \Delta\Lambda)^{-1}) = (233\,\mathrm{s}, 847\,\mathrm{s})$.

We find that the mixed exponential distribution improves the fit at the tail in Fig. 0.2. Though it is not statistically favored over the simple exponential model in the Bayesian sense, the new parameters are informative about chronological aspects of the data. In comparison, the Weibull distribution, $p(t) = k/\Lambda(t/\Lambda)^{k-1}e^{(t/\Lambda)^k}$, where the rate of decay changes as t^{k-1}, is hardly an improvement over the simple exponential model, and we recover the same parameters as the exponential fit ($\Lambda = 2.4 \times 10^{-3}\,\mathrm{s}^{-1}$, $k = 1.01$).

We were motivated to mix many exponentials because the average peace duration shortens over the course of the day, so we plot our maximum likelihood fit against the binned averages ordered chronologically in Fig. 0.2. Our maximum likelihood parameters were not constrained to fit the chronological order of the data, but when plotted as a function of ordered data they match a linear fit to the shown points closely. The nontrivial prediction of the ordering of the data suggests that the peace distribution is really a nonstationary exponential process that evolves over the day.

The nonstationary exponential model contains two natural time scales for describing the outbreak of fighting. The slowly changing parameter $\Lambda(t)$ typically curves on the order of hours, reflecting endogenous change in aggression from time spent in the compound and exogenous effects from the environment like temperature and shade (data not shown). Within any particular hour of the day, the local time scale is between 230 s and 850 s, the former defining a typical lower bound on the timescale on which two sequential episodes of conflict become distinct.[1]

0.3 Scaling of arithmetic variance

Assuming that the distribution of fight durations conditioned on fight size n can be written in a scale free form as

$$p_{\mathrm{n}}(t) = \frac{1}{\mu_{\mathrm{n}}}\phi\left(\frac{t}{\mu_{\mathrm{n}}}\right) \tag{2}$$

corresponds to a perfect scaling collapse of the form suggested by Fig. 2. In this case, the geometric variance is constant, and the arithmetic variance $\sigma^2 \propto \mu_{\mathrm{n}}^2$. Because $\tilde{\mu}_{\mathrm{n}} \propto \mu_{\mathrm{n}}$, this implies $\sigma^2 \propto \tilde{\mu}_{\mathrm{n}}^2$. In Fig. S3, we show how the arithmetic mean scales with fight size.

Similar scale free forms for growth processes have been found in distributions of votes in elections (*3*), size distributions of unicellular eukaryotes (*4*), bacterial cell sizes in varying conditions (*5*), and more generally under the name of fluctuation scaling in a variety of systems

[1] It is important that this timescale is well above the 120 s criterion used by JC Flack to demarcate the end of a conflict episode (Supplementary Materials, Sec. 0.1.2.)

(*6*). Additionally, growth processes produce distributions well-approximated by lognormals when they are the result of many independent multiplicative factors (by the analogue of the Central Limit Theorem in log-space). Below, we argue for a different mechanism in our system: our scaling collapse results from pairwise fight durations having a nearly lognormal distribution and larger conflicts consisting of correlated sums of pairwise durations.

0.4 Diffusion models

The scaling collapse with a universal lognormal curve suggests that the standard deviation is proportional to the mean: $\sigma \propto \tilde{\mu}$. As we show in Fig. S5, however, many of the measured variances fall below the line and weighted least squares returns a sublinear relationship, $\sigma \propto \tilde{\mu}^{0.95}$. If conflict duration is explained as the sum of $\alpha\binom{n}{2}$ random variables representing the duration of pairwise interactions then this scaling implies that the interactions are correlated in duration, but the correlation is not perfect (Table S1). If they were uncorrelated, we would observe $\sigma \propto \tilde{\mu}^{1/2}$. Here, we describe the two different diffusion-drift models we use to measure the deviation from perfect correlation.

We propose a random walk model where a single conflict consists of a sequence of pairwise interactions each with some duration t_k at the kth interaction. In the limit that the duration of all interactions in a sequence are perfectly correlated, $t_k = t_j$ for all pairs (j, k), the correlation time is infinite and the probability distribution of the first interaction $p(t_0)$ should be consistent with the observed distribution $p_2(t)$ so that we recover the observed lognormal distribution (Eq 1). In the limit where each sequential interaction is completely independent of the past, the distribution at each time step should be consistent with $p_2(t)$ and consequently the equilibrium distribution $p(t_{k\to\infty}) = p_2(t)$, but this leaves open the question of the temporal dynamics that lead to it.

A simple and solved set of dynamics that converges to the observed lognormal distribution

Table S1: Hypotheses for conflict duration. Fight durations obey a simple scaling law in which the durations of fights of size n are rescaled versions of smaller fights, by a factor that scales as $\binom{n}{2}$. This growth with the number of pairs suggests that the durations of fights of size greater than 2 may arise from a process consisting of multiple pairwise interactions, each with duration T_2 sampled from the duration distribution for fights of size 2. In this table, we compare the outcomes of some possible processes. First, branching models are inconsistent with the data because the number of interactions, and thus the mean duration, grows too slowly with n. Second, sequentially adding a fraction of possible pairwise interactions ("pairwise sequential") produces the correct scaling of the mean, but insufficiently large standard deviation. Finally, sequentially adding correlated versions of pairwise durations can produce the correct scaling of the first two moments of the duration distribution. Only the perfect correlation case obeys an exact rescaling, but our data cannot distinguish between the "correlated" and "perfectly correlated" cases.

Model	Schematic for fight of size 5	Mean	Variance	Self-similar	Fits data
Branching simultaneous	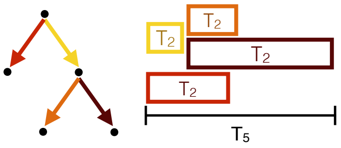	μ sub-linear in n			✗
Branching sequential	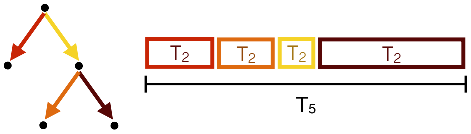	$\mu \propto n$	$\sigma^2 \propto \mu$		✗
Pairwise sequential	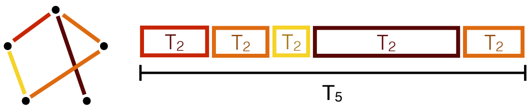	$\mu \propto \binom{n}{2}$	$\sigma^2 \propto \mu$	No	✗
Pairwise sequential, perfectly correlated	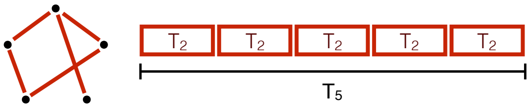	$\mu \propto \binom{n}{2}$	$\sigma^2 \propto \mu^2$	Yes	✓
Pairwise sequential, correlated	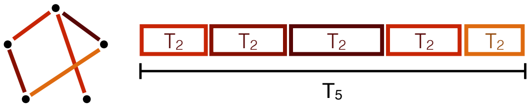	$\mu \propto \binom{n}{2}$	$\sigma^2 \propto \mu^c$, $1 < c < 2$	Nearly	✓

corresponds to diffusion in logarithmic space, or the Ornstein-Uhlenbeck process in statistics and more commonly known as the Fokker-Planck equation in statistical physics (*9, 10*). Besides having the proper stationary distribution, it has a single parameter D that controls how quickly correlations disappear in sequential interactions. Generally speaking, different dynamics will lead to differently shaped curves for decorrelation, but we are primarily interested in the characteristic decay time for correlations which determines the scaling between the variance and the means. Moreover, we show that we find similar results for another model with different dynamics later.

In the Fokker-Planck model (FPM), the initial interaction takes some duration t_0 with distribution $p_2(t_0)$ and the next duration is given by a random multiplicative factor ξ_1 that multiplies the duration of the previous interaction $t_1 = \xi_1 t_0$. This corresponds to a random walk in logarithmic space. The variance of the multiplicative factor is controlled by a diffusion constant D that determines how quickly sequential pairwise interactions decorrelate. The corresponding differential equation for the movement of the random walk (during a *single* conflict) in log-duration space ($\eta = \ln t/\tilde{\mu}_2$) is

$$\partial_k p(\eta, k) = \gamma \partial_\eta [\eta\, p(\eta, k)] + D \partial_\eta^2 p(\eta, k) \tag{3}$$

where k corresponds to the kth pairwise interaction in the sequence. The ratio D/γ determines how quickly the random walker returns to the equilibrium distribution.

The stationary distribution is given by

$$p(\eta, k \to \infty) = \sqrt{\frac{\gamma}{2\pi D}} e^{-\gamma\eta^2/2D}. \tag{4}$$

Thus, the parameter γ is fixed by the observed geometric variance $D/\gamma = \tilde{\sigma}^2$, and there is only one parameter that determines how quickly correlations decay.

The correlation of the duration of sequential pairwise interactions is the average over all

initial starting conditions and all possible trajectories given an initial starting duration t_0

$$\langle t_0 t_k \rangle - \langle t_0 \rangle^2 = \int_0^\infty dt_0 \, t_0 \, p_\infty(t_0) \int_0^\infty dt_k \, t_k \, p(t_k|t_0) - e^{2\ln\tilde{\mu_2}+\tilde{\sigma}^2} \tag{5}$$

$$= e^{2\ln\tilde{\mu}_2+\tilde{\sigma}^2} \left(e^{\tilde{\sigma}^2 e^{-kD/\tilde{\sigma}^2}} - 1 \right), \tag{6}$$

where the transition probability $p(t_k|t_0)$ is the Greens function for the Orstein-Uhlenbeck process. Normalizing Eq 6 we find that the correlation in duration of sequential pairwise interactions decays with k as

$$\chi(k) = \left(e^{\tilde{\sigma}^2 e^{-kD/\tilde{\sigma}^2}} - 1 \right) \Big/ \left(e^{\tilde{\sigma}^2} - 1 \right). \tag{7}$$

We take the unitless characteristic decay time k^* to be the number of interactions it takes for χ to decay to $1/e$:

$$k^* = \frac{\tilde{\sigma}^2}{D} \left[\ln \tilde{\sigma}^2 - \ln \left(\ln \left(\frac{e^{\tilde{\sigma}^2} - 1}{e} + 1 \right) \right) \right]. \tag{8}$$

To get some intuition for this expression, we expand around small $\tilde{\sigma}^2$, finding

$$\chi(k) \approx e^{-kD/\tilde{\sigma}^2} \tag{9}$$

in which case

$$k^* \approx \tilde{\sigma}^2 / D \tag{10}$$

As we expect, a larger diffusion coefficient means that correlations decay faster.

Given that the (arithmetic) average pairwise fight has duration $\mu_2 = 10.0\,\mathrm{s}$, we define a decorrelation time in units of seconds as

$$\tau := \mu_2 k^*. \tag{11}$$

We find that the data is consistent with $D < 0.0175$, or $\tau > 270\,\mathrm{s}$, well beyond the typical duration of a conflict.

The distribution of conflict durations at the kth interaction is the convolution of all distributions up to $k-1$.

In an alternative set of dynamics that we consider (the Probability Density Model, or PDM), we imagine that we sample interactions from the data, where we choose we choose data points more similar to previous ones in low diffusion and more randomly in high diffusion. More formally, let x be a unitless "aggression" variable that varies from 0 to 1, and corresponds to the cumulative probability that the duration of a pairwise interaction is less than x: as a function of interaction duration t_k,

$$x_k = x(t_k) = \int_0^{t_k} p_2(t)\, dt. \tag{12}$$

The diffusion model does a random walk in x-space. At each step, the probability of moving a distance δx in x-space is a Gaussian with variance D, with reflecting boundaries so that the step does not take x outside $[0, 1]$.

This model is expensive to simulate and difficult to analyze analytically. Instead of computing the shape of the full probability distribution like we do with the FPM, we approximate the distributions by simulating a set of trajectories. We compare the samples with the Kolmogorov-Smirnov test in Fig. S7 over and plot the cumulative error between the data and approximate CDFs in Fig. S8. We find similarly as with the FPM that characteristic decay times $\tau \gtrsim 10^2\,\mathrm{s}$ return similar fits to the data. The results of the tests are summarized in Fig. S7, and the corresponding decorrelation times are shown in Fig. S9.

0.5 Predicting conflict growth

A crucial question for managing conflict is estimating the size and duration to which an ongoing conflict might grow. Accurate predictions of these quantities could help decide when to allocate limited resources to limit or even promote conflict. Using our model, we can make predictions of how many more individuals will join an ongoing fight and how long we can expect the

conflict to last.

In the previous section, we have shown how our model captures the distribution of conflict duration conditional on the conflict size $p(t|n)$, and we combine this with the observed $p(n)$ to model the full distribution $p(t,n) = p(t|n)p(n)$. The probability that a fight will extend by time Δt with Δn additional members given that we have observed n_0 participants at time t_0 is an application of Bayes' theorem:

$$p(\Delta t, \Delta n|t_0, n_0) = \frac{p(t_0, \Delta t, n_0, \Delta n)}{p(t_0, n_0)} \tag{13}$$

$$= \frac{p(t = t_0 + \Delta t, n = n_0 + \Delta n)}{p(t_0, n_0)} \tag{14}$$

$$= \frac{p(t|n)p(n)}{\sum_{\Delta n} \int p(t_0 + \Delta t|n_0 + \Delta n)p(n_0 + \Delta n)\, d\Delta t} \tag{15}$$

where we have used the fact that the full duration of the conflict $t = t_0 + \Delta t$ and the final size of the conflict $n = n_0 + \Delta n$.

By marginalizing out either Δt or Δn, we can focus on how large a conflict might become or how long it might last:

$$p(\Delta n|n_0, t_0) = \int p(\Delta t, \Delta n|t_0, n_0)\, d\Delta t \tag{16}$$

$$p(\Delta t|n_0, t_0) = \sum_{\Delta n} p(\Delta t, \Delta n|t_0, n_0) \tag{17}$$

We show an example of a dyadic $n_0 = 2$ conflict that has been ongoing for $t_0 = 10$ s in Fig. S10. In this particular example, the conflict is more likely to grow to size 3 or 4 before resolving, reflecting the fact that most conflicts of size 2 are less than 6.7 s. On average, this fight will grow by 2.5 individuals but with rather large standard deviation of 2.8. The average time extended is (63 s) with standard deviation (177 s), the large standard deviation reflecting skewed statistics from a heavy-tailed distribution.

We can compare potential consequences of stopping the conflict at particular times. In Fig.'s S11 and S12, we consider interventions that stop an ongoing fight between two individuals

that has progressed for $2\,\mathrm{s}$, $10\,\mathrm{s}$, and $20\,\mathrm{s}$.

One possible goal is to minimize total conflict size. Here, an early intervention at $t_0 = 2\,\mathrm{s}$ does not prevent as many individuals from joining as an intervention at $t_0 = 10\,\mathrm{s}$ when the probability of another individual joining grows to a factor of 2 above the probability of no other individuals joining. At $t_0 = 20\,\mathrm{s}$ it is 3x more likely that another individual will eventually join.

Another objective could be to control the total duration at the time of observation t_0, but this scenario again depends on the particular risk we wish to minimize. If maximizing the probability that the fight ends immediately, we note fights are relatively most likely to end immediately sometime between $t_0 = 2\,\mathrm{s}$ and $t_0 = 20\,\mathrm{s}$ when it might make more sense to wait for the conflict to end rather than intervening. On the other hand, given a single opportunity to intervene, the most consequential intervention on average is at $20\,\mathrm{s}$ because the average duration extended is $47 \pm 145\,\mathrm{s}$, $63 \pm 177\,\mathrm{s}$, and $87 \pm 215\,\mathrm{s}$, respectively; thus, a late intervention causes the most change in the expected duration of conflict. Averages, however, are skewed for heavy-tailed distributions. Indeed, this dominance of the tail beyond $20\,\mathrm{s}$ suggests that a risk-averse objective—minimizing the possibility of long conflicts—would involve stopping conflict early.

If only a limited number of interventions are available for a large number of conflicts, mitigating total time spent in conflicts or the number of participants would involve choosing intervention times order for maximum effect. In this particular scenario, efficient use of intervention opportunities may be an identifiable strategy applied by conflict managers in social systems.

0.6 Human conflict

We look at how the geometric mean of human armed conflict durations scales with the number of participating entities using two openly available datasets: the Correlates of War (COW) and Uppsala Conflict Data Program (UCDP) databases (*11–13*).

The COW database only includes wars that "must involve sustained combat, involving orga-

nized armed forces, resulting in a minimum of 1,000 battle-related combatant fatalities within a twelve month period" as well as be between entities that can provide "effective resistance" (*12*). As we show in Fig. S13, the geometric means for conflicts with $n \geq 3$ follow a binomial scaling better than a simple linear scaling as with the macaque conflict data. We compare the linear and binomial models by assuming a lognormal distribution for each conflict size with a mean given by the model with some unknown universal variance $\tilde{\sigma}^2$. When we take the log-likelihood ratio R of the binomial model to the linear model, the variance cancels out, and $R = 9.0$ for $N = 29$ conflicts of size $n \geq 3$.

One clear anomaly that we do not consider for our fit is the distribution of wars between only two states. Here, the distribution is much wider compared to larger wars, and the geometric mean is higher than for conflicts with 3, 4, or 6 states. It is not clear why conflicts for $n = 2$ do not follow the trend established by larger conflicts. One possibility is that pairwise interstate conflicts are different. Other possibilities may have to do with the classification of pairwise conflicts or how the duration is determined.

The UCDP database counts conflict episodes "defined as years of continuous use of armed force in a conflict" (*13*) where an armed conflict is "a contested incompatibility that concerns government and/or territory where the use of armed force between two parties, of which at least one is the government of a state, results in at least 25 battle-related deaths in a calender-year" (*11*). Here, it is clear that there is a discrepancy in the growth of the mean after $n \gtrsim 10$. Fitting to conflicts $3 \leq n \leq 10$, we find that the log-likelihood ratio $R = 1.0$. This means that for small conflicts there is insufficient evidence to show that one scaling is a better explanation than the other.

That the scaling of means with size follows a similar pattern at all is highly intriguing because the UCDP dataset includes a variety of conflicts between states and between states and non-states within shared territory or even on outside territories. Furthermore, the definition of

conflict and duration differ between the COW and UCDP databases. Evidently, other details are important for comparing analogous human conflicts with the macaque system on which we focus, but it seems that similar scaling ideas may provide a place to start for comparison across these systems.

References and Notes

1. J. Caldecott, *An Ecological and Behavioral Study of the Pigtailed Macaque* (Karger, 1986).
2. J. C. Flack, *Philosophical Transactions of the Royal Society B: Biological Sciences* **367**, 1802 (2012).
3. S. Fortunato, C. Castellano, *Physical Review Letters* **99**, 138701 (2007).
4. A. Giometto, F. Altermatt, F. Carrara, A. Maritan, A. Rinaldo, *PNAS* **110**, 4646 (2013).
5. S. Taheri-Araghi, *et al.*, *CURBIO* **25**, 385 (2015).
6. Z. Eisler, I. Bartos, J. Kertész, *Advances in Physics* **57**, 89 (2008).
7. M. Newman, *Contemporary Physics* **46**, 323 (2005).
8. A. Clauset, C. R. Shalizi, M. E. J. Newman, *SIAM Review* (2009).
9. H. Risken, *The Fokker-Planck Equation* (1984).
10. N. G. Van Kampen, *Stochastic Processes in Physics and Chemistry* (Elsevier, 2011).
11. N. P. Gleditsch, P. Wallensteen, M. Eriksson, *Journal of Peace Research* (2002).
12. M. R. Sarkees, F. W. Wayman, *Resort to war: a data guide to inter-state, extra-state, intra-state, and non-state wars, 1816-2007*, Correlates of war series (CQ Press, 2010).
13. J. Kreutz **47**, 243 (2010).

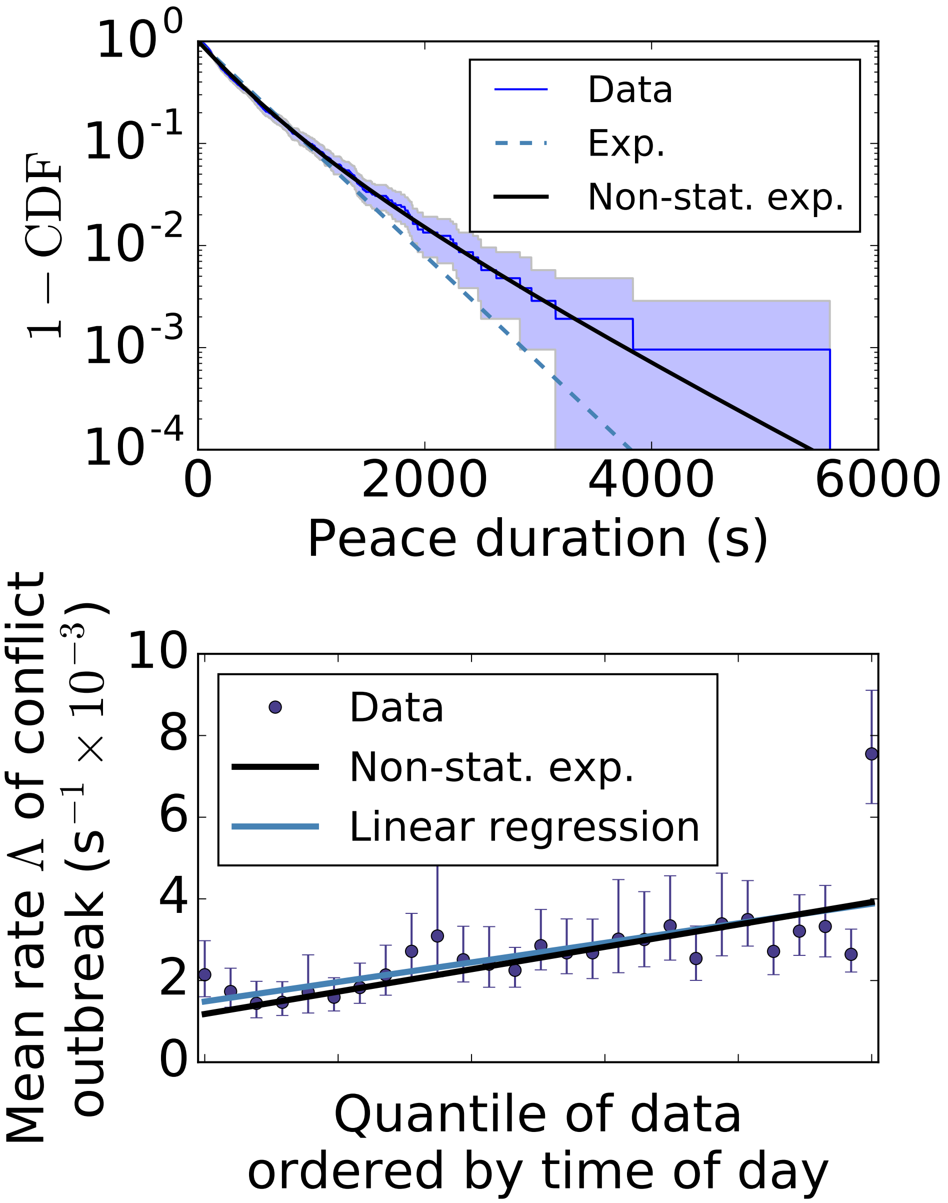

Figure S2: Peaceful periods. (top) Distribution of peace bout duration. Maximum likelihood exponential (dashed) finds the typical duration of peaceful periods to be $1/\Lambda \approx 400\,\mathrm{s}$. Mixed exponential distribution across a uniform distribution of parameters (black) fits the tail and gives a range of time scales from $233\,\mathrm{s}$ to $847\,\mathrm{s}$. (bottom) Change in rate of conflict outbreak ordered from earlier to later in the observational day. Last point shows a strong tendency for conflict to erupt in the moments before the macaques are removed from the compound, but note that it is the mean of 31 observations compared to 39 data points for the other points. Mixed exponential fit assumes no ordering to the data, but when plotted by time of day (black) it predicts the linear trend. We compare its slope of $2.7 \times 10^{-3}\,\mathrm{s}^{-1}$ per quantile to a linear regression (blue) with slope $2.4 \times 10^{-3}\,\mathrm{s}^{-1}$. Error bars are 90% confidence intervals from bootstrapped means.

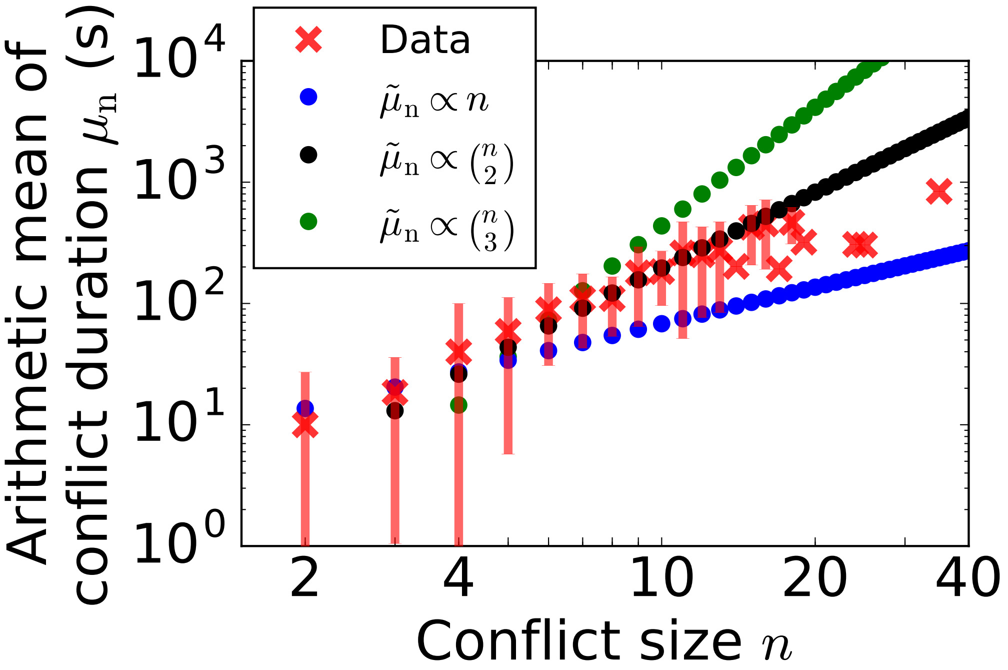

Figure S3: Scaling of the arithmetic means of conflict duration with fight size with error bars spanning two standard deviations. See Fig. 1 for analogous plot with geometric means.

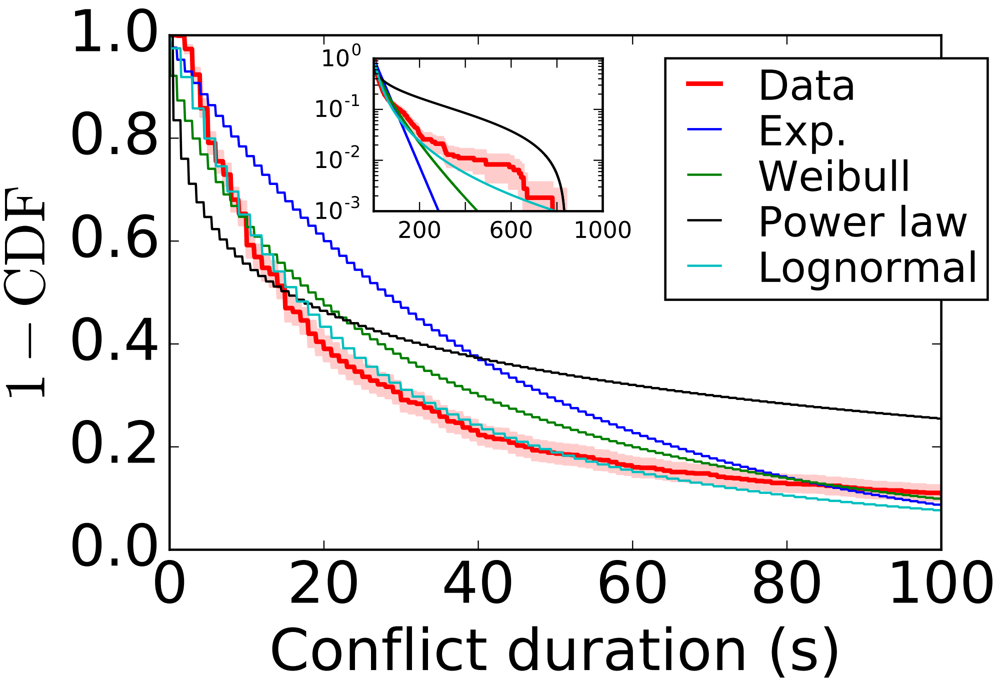

Figure S4: Fits to aggregate distribution of conflict duration. Maximum likelihood fits of several common families of distributions to observed conflict durations. Exponentially decaying distributions like the Weibull cannot fit the heavy tail. Note that on a logarithmic plot, deviations in the tail are highly conspicuous (*7, 8*).

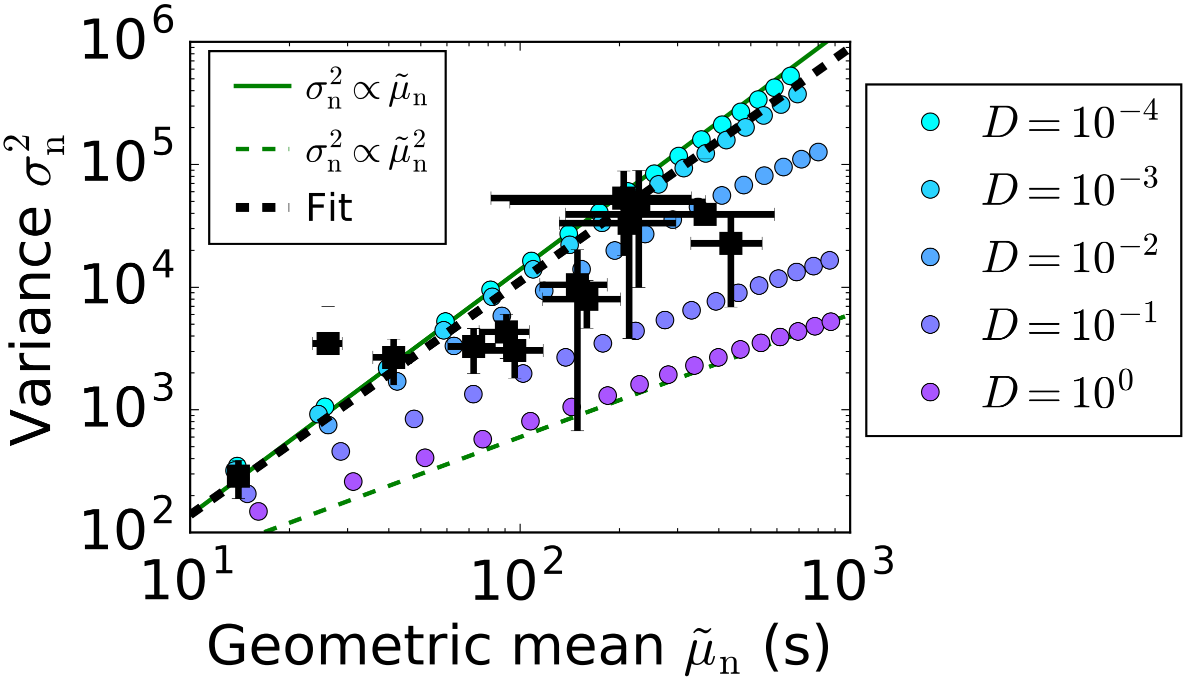

Figure S5: PDM scaling of variance with geometric mean as a function of diffusion constant D. Data follows $\sigma_n^2 \propto \tilde{\mu}_n^{1.9}$ (dashed black), significantly faster scaling than the completely uncorrelated case where $\sigma_n^2 \propto \tilde{\mu}_n$ (dashed green), but not quite the perfectly correlated case $\sigma_n^2 = \tilde{\mu}_n^2 e^{\tilde{\sigma}^2}(e^{\tilde{\sigma}^2} - 1)$ (solid green). Weighted linear fit has slope between with $D = 10^{-3}$ and $D = 10^{-4}$ but data seems to also agree with $\tau \gtrsim 10^2$ s, consistent with the results from the FPM suggesting $\tau > 270$ s (Fig. S6).

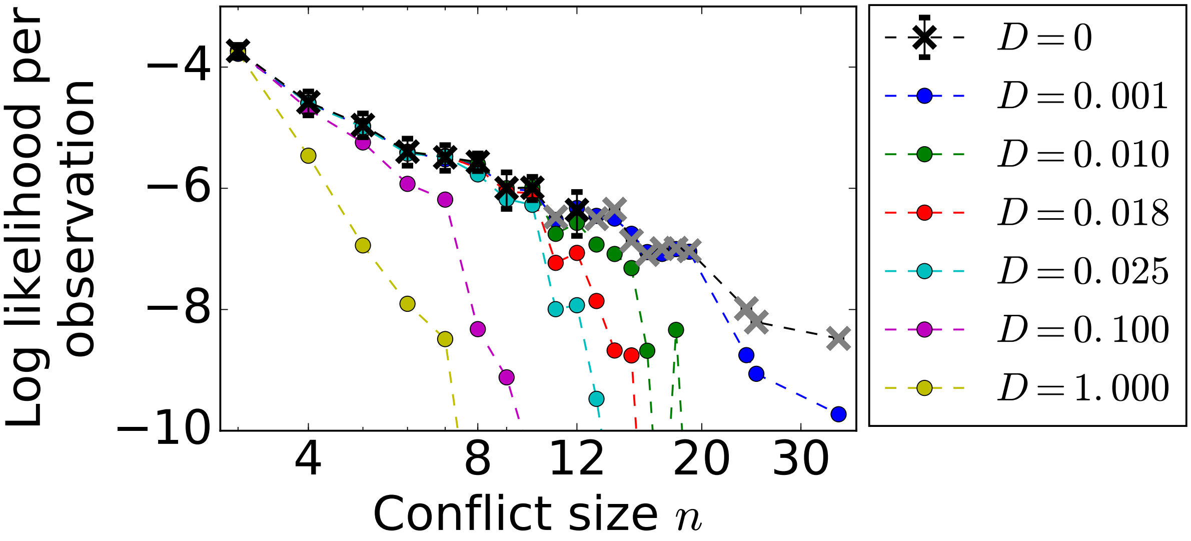

Figure S6: Log-likelihood per observed conflict given conflict size for FPM. Infinite correlation time with diffusion coefficient $D = 0$ maximizes likelihood, but models are indistinguishable for $D < 0.0175$ ($\tau > 270\,\mathrm{s}$) given error bars that are 90% confidence intervals from bootstrapped data. Conflict sizes with only one observation are denoted with grey x's. By combining all observations for $n \geq 11$ and doing another comparison of the distribution of log-likelihoods with the KS-test, we again find significant differences for $D \geq 0.0175$, confirming our lower bound.

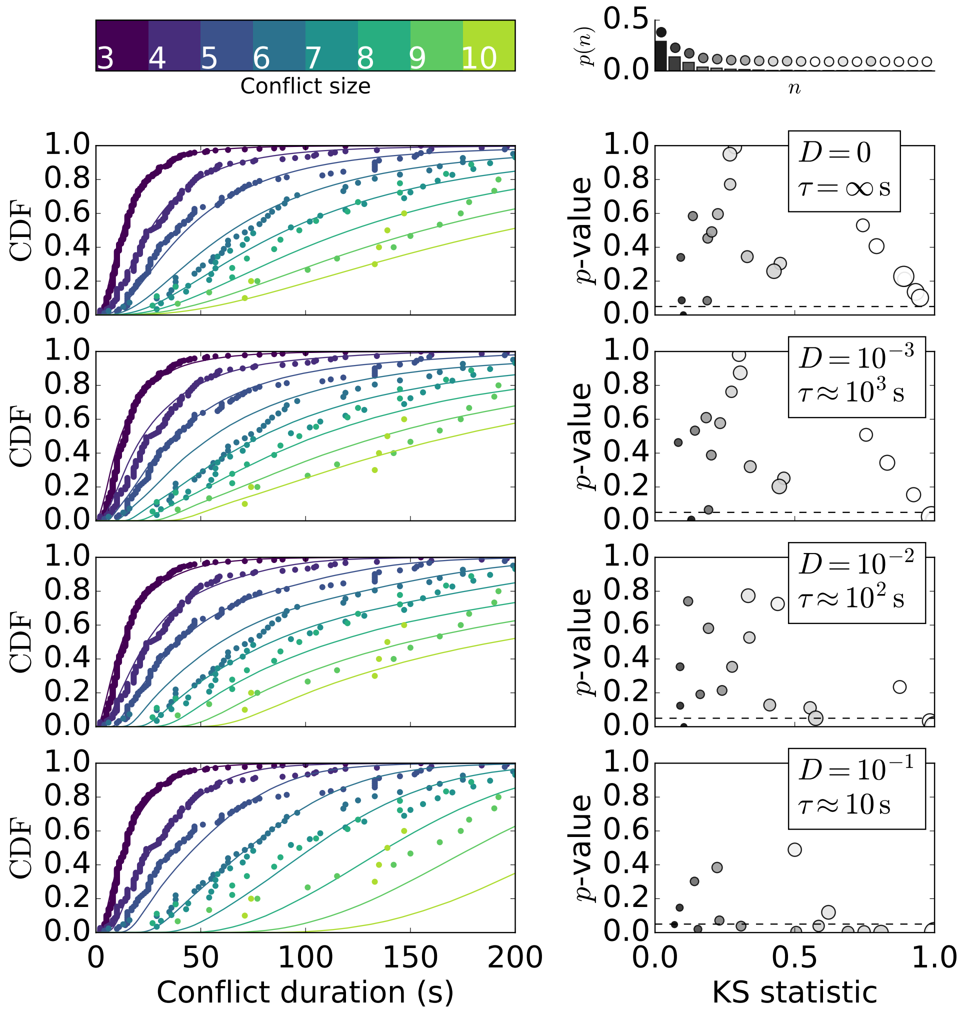

Figure S7: PDM fits to data. (left) PDM prediction of distribution of conflict duration. Conflict sizes go from 3–10 from black to green. Data as circles and model predictions are connected by a line. (right) KS test statistics and p-values, where the size of the circle is proportional to the conflict size and the color represents relative frequency of the conflict size (top). Dashed line demarcates $p \leq 0.05$. Fits are within expected fluctuations for $D = 0$ and continue to be reasonable for $D \leq 10^{-3}$. See Fig. S8.

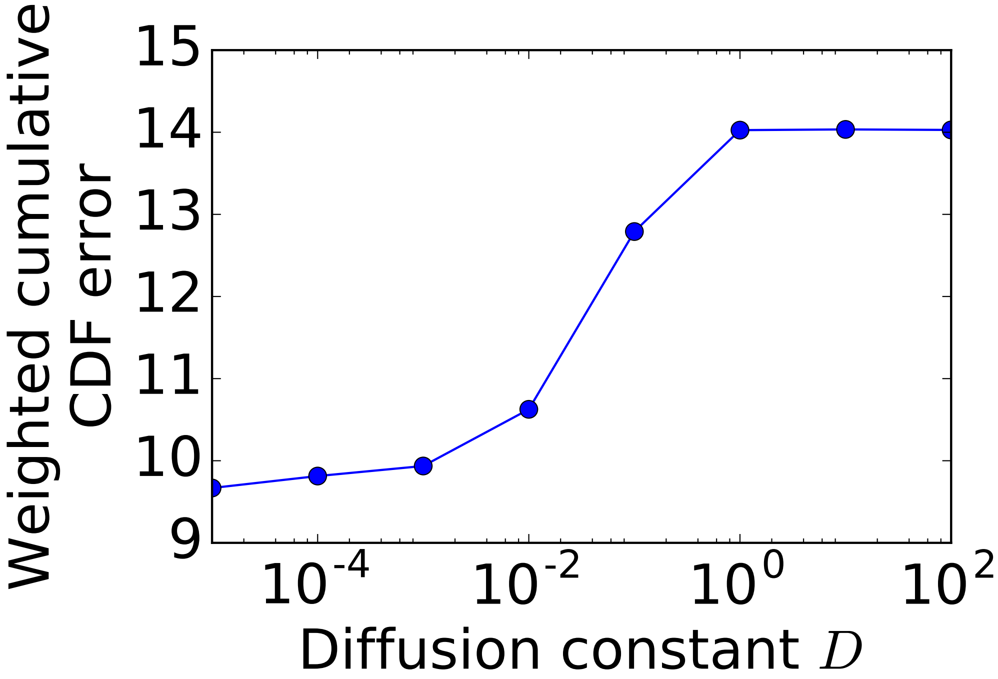

Figure S8: Cumulative CDF error between data and PDM. The cumulative error is the integral of the absolute difference in logarithmic space weighted by the fraction of data for a conflict of size n: $\sum_n p_n \int_{-\infty}^{\infty} |\mathrm{CDF}_{\mathrm{data}}(t) - \mathrm{CDF}_{\mathrm{model}}(t)|\, d\ln t$. Cumulative error starts to plateau between $D = 10^{-2}$ and $D = 10^{-3}$ corresponding to a decorrelation time of $100\,\mathrm{s} < \tau < 1000\,\mathrm{s}$ (Fig. S9), in agreement with the more precise measurements from the FPM in Fig. S6.

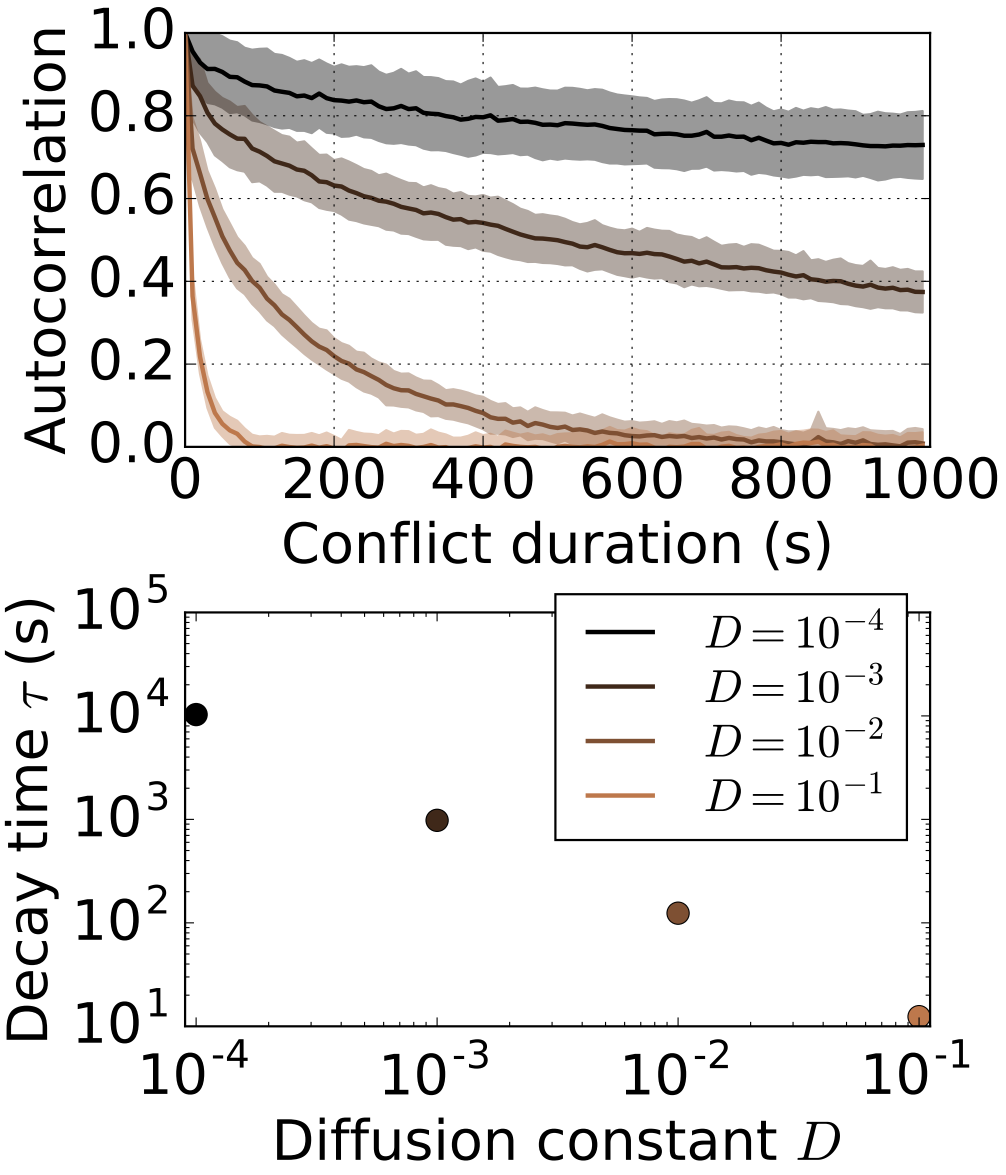

Figure S9: PDM decorrelation time as a function of diffusion constant D using the average pairwise interaction duration $\mu_2 = 10.0\,\text{s}$ as the scale.

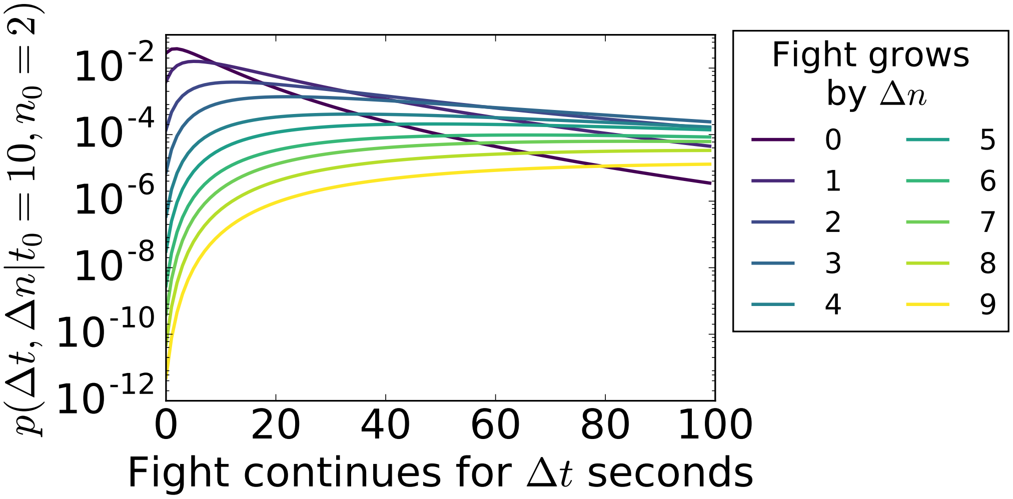

Figure S10: Probabilities $p(\Delta t, \Delta n | t_0 = 10, n_0 = 2)$ of an ongoing fight with 2 individuals after 10 s continuing for Δt seconds and Δn more participants. We use the lognormal model of fight duration instead of the empirical distributions and use the empirical distribution of fight sizes up to 18 (although we only show $\Delta n < 10$).

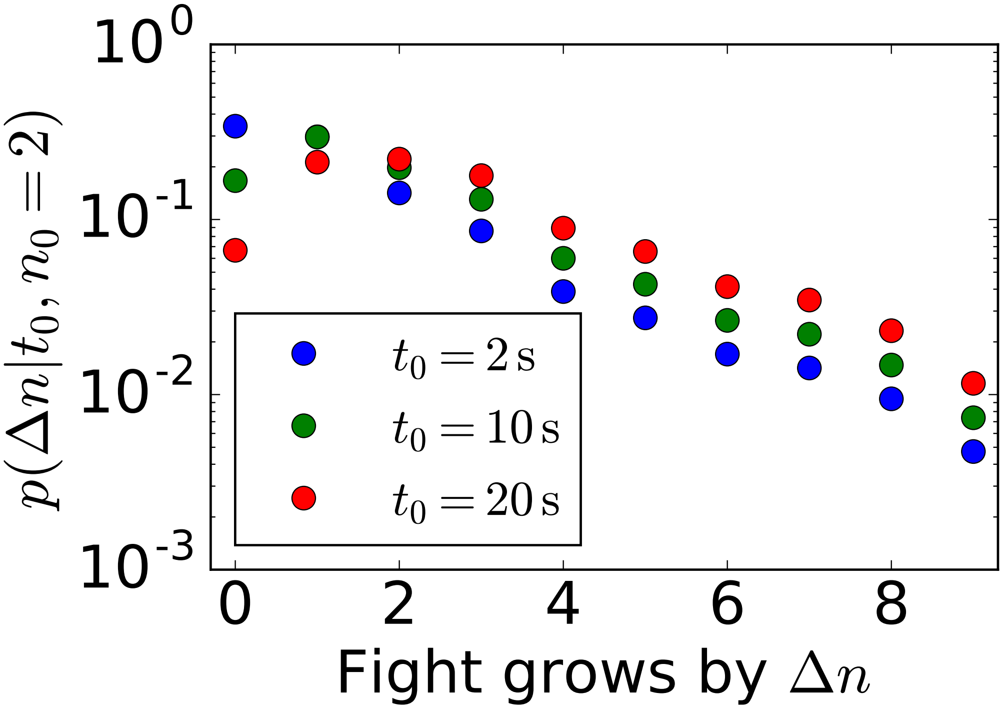

Figure S11: Probabilities $p(\Delta n | t_0, n_0 = 2)$ that fights with 2 individuals still ongoing at 2 s, 10 s, and 20 s grow by Δn more participants as from Eq 16.

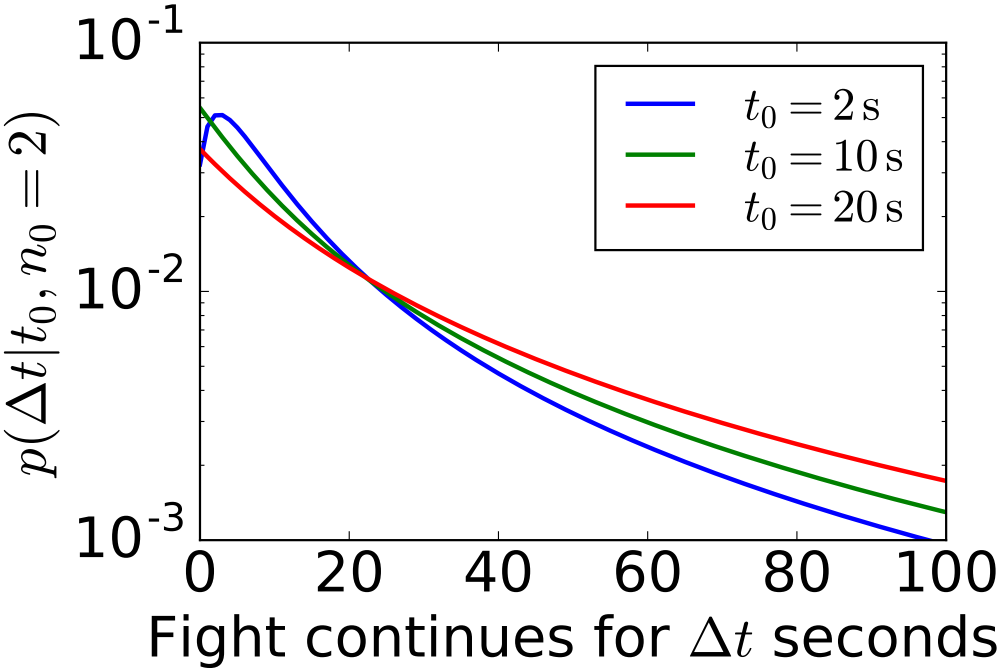

Figure S12: Probabilities $p(\Delta t|t_0, n_0 = 2)$ that fights with 2 individuals still ongoing at 2 s, 10 s, and 20 s last for Δt more seconds as from Eq 17.

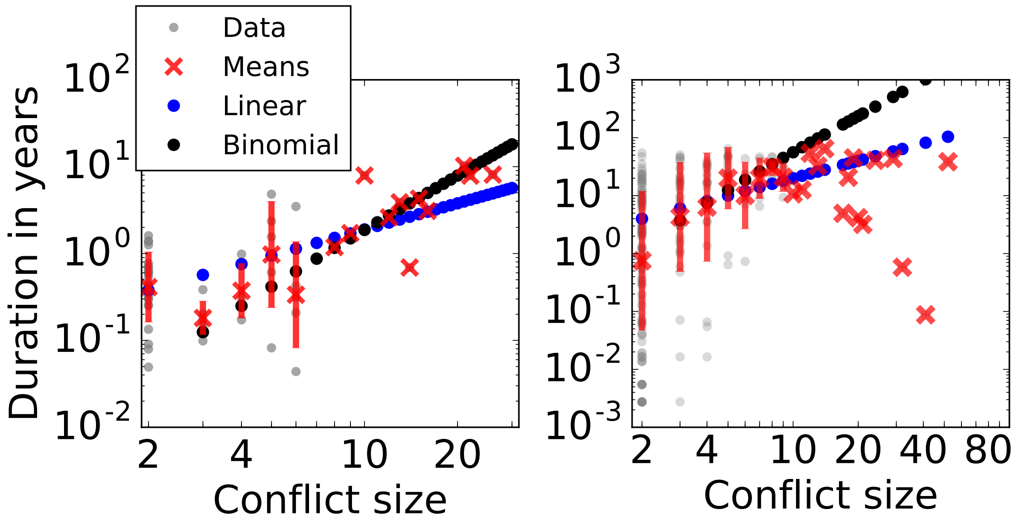

Figure S13: Scaling of geometric means of interstate wars. (left) COW database and (right) UCDP. The COW means scale as $\binom{n}{2}$ like with the macaque conflict data for $n \geq 3$, and the scaling of the UCDP means is compatible with $\binom{n}{2}$ growth.
